# Effect of Biochar Amendment and Ageing on Adsorption and Degradation of Two Herbicides

**DOI:** 10.1007/s11270-017-3392-7

**Published:** 2017-05-25

**Authors:** Alena Zhelezova, Harald Cederlund, John Stenström

**Affiliations:** 1 Soil Biology Department, Soil Science Faculty, Moscow State University, Leninskie Gory 1-12, Moscow, Russian Federation 119991; 2 V.V. Dokuchaev Soil Science Institute, Pyzhyovskiy lane 7 building 2, Moscow, Russian Federation 119017; 3 Department of Molecular Sciences, Uppsala BioCenter, Swedish University of Agricultural Sciences (SLU), Box 7015, 750 07 Uppsala, Sweden

**Keywords:** Biochar, Glyphosate, Diuron, Herbicide degradation, Adsorption, Biochar ageing

## Abstract

Biochar amendment can alter soil properties, for instance, the ability to adsorb and degrade different chemicals. However, ageing of the biochar, due to processes occurring in the soil over time, can influence such biochar-mediated effects. This study examined how biochar affected adsorption and degradation of two herbicides, glyphosate (N-(phosphonomethyl)-glycine) and diuron (3-(3,4-dichlorophenyl)-1,1-dimethylurea) in soil and how these effects were modulated by ageing of the biochar. One sandy and one clayey soil that had been freshly amended with a wood-based biochar (0, 1, 10, 20 and 30% *w*/*w*) were studied. An ageing experiment, in which the soil-biochar mixtures were aged for 3.5 months in the laboratory, was also performed. Adsorption and degradation were studied in these soil and soil-biochar mixtures, and compared to results from a soil historically enriched with charcoal. Biochar amendment increased the pH in both soils and increased the water-holding capacity of the sandy soil. Adsorption of diuron was enhanced by biochar amendment in both soils, while glyphosate adsorption was decreased in the sandy soil. Ageing of soil-biochar mixtures decreased adsorption of both herbicides in comparison with freshly biochar-amended soil. Herbicide degradation rates were not consistently affected by biochar amendment or ageing in any of the soils. However, glyphosate half-lives correlated with the Freundlich Kf values in the clayey soil, indicating that degradation was limited by availability there.

**Electronic supplementary material:**

The online version of this article (doi:10.1007/s11270-017-3392-7) contains supplementary material, which is available to authorized users.

## Introduction

Biochar is a carbon-rich material created by pyrolysis, i.e. incomplete combustion of biomass at temperatures between 200 and 800 °C under limited presence of oxygen (Kookana 2010). Biochar can be made from different organic substrates (wood, straw, manure) using a wide range of pyrolysis conditions, which results in highly variable properties. The feedstock usually determines the chemical composition, quantity of macropores and nutrient content in biochar. Pyrolysis conditions (time, temperature, pressure) determine the morphology and surface structure changes in feedstock and C/H content (Ahmad et al. 2014). There is currently great interest in using biochar application for improving the properties of agricultural soils, as it generally reduces soil acidity and increases soil porosity and water-holding capacity. Other potential effects of biochar amendment to soil include carbon sequestration and reduction of greenhouse gas emissions, improved soil fertility, plant growth promotion and sorption and deactivation of agrochemicals (Ahmad et al. 2014; Biederman and Harpole 2013; Cayuela et al. 2014; Jeffery et al. 2011).

The high capacity of biochars to bind pollutants makes them useful for remediation of urban soils, wastelands and wastewaters (Beesley et al. 2011; Herath et al. 2016; Kookana 2010). Biochar can non-selectively adsorb different types of organic and inorganic chemicals because of its large surface area and high porosity (Herath et al. 2016). In addition, specific adsorption sites on the biochar surface, such as the aromatic-rich core structure and oxidised surface groups with variable charge and hydrophobicity, can lead to more selective interactions with herbicides. Biochar-mediated increases in adsorption to soil have been observed for many herbicides, for example diuron (Yang et al. 2006), bromoxyl and ametryne (Sheng et al. 2005), simazine (Jones et al. 2011) and MCPA (Tatarková et al. 2013).

Although soil amendment by biochar is a promising technique for pollutant retention, biochar may lose some of its beneficial properties due to ageing. Ageing is a combination of several processes occurring after the biochar has been incorporated into the soil that may have contrasting effects on its adsorption properties. On the one hand, these processes can lead to increased adsorption, as oxidation of exposed C rings with high density of π electrons leads to the introduction of oxygen-containing functional groups on the biochar surface (Joseph et al. 2010). Changes in surface charge, with an increase in cation exchange capacity, are usually observed as a consequence of ageing (Cheng et al. 2008). Degradation of hydrophobic materials that have condensed on the biochar surface during pyrolysis leads to an increase in porosity and adsorption of some pesticides (Trigo et al. 2014). On the other hand, adsorption of labile organic compounds and soil mineral particles may block access to many of the adsorption sites. Minerals may be adsorbed on the surface of biochar, due to presence of carboxylic and phenolic functional groups introduced by oxidation and of previously adsorbed soil organic matter (Lin et al. 2012). Such processes have the potential to reduce adsorption of pesticides.

In fact, while addition of fresh biochar to soil generally increases herbicide sorption, a significant decrease in adsorption is a common result of the biochar ageing process (Hale et al. 2011; Martin et al. 2012; Zhang et al. 2016). For example, atrazine adsorption in soil amended with biochar 32 months earlier was similar to that in control soil (Martin et al. 2012). Thus, ageing may limit the usefulness of biochar application for remediation purposes (Zhang et al. 2016). However, there is evidence that in some cases, biochar can serve as an effective sorbent of herbicides (indaziflam and fluoroethyldiaminotriazin) for at least 2 years (Trigo et al. 2014).

Biochar amendment also affects the degradation of herbicides in soil in several ways and the effects can be either stimulatory or suppressive. Biochar may contain available nutrients that stimulate overall microbial activity and thus degradation (Jablonowski et al. 2013; Safaei Khorram et al. 2016). However, degradation of herbicides in biochar-amended soils is most commonly reduced because herbicide adsorption increases (Beesley et al. 2011). Biochar also sorbs dissolved organic carbon, which can contribute to co-metabolic biodegradation (Lin et al. 2012). Some changes in the degradation rate can be a result of indirect effects of biochar amendment, e.g. changes in soil pH, albedo and aeration.

The aim of this study was to investigate how the effects of biochar on adsorption and microbial degradation of herbicides in soil are modulated by herbicide type, soil type and ageing processes. Two herbicides (glyphosate and diuron) were chosen because of their contrasting chemical properties and prior knowledge about differences in their sorption affinity for soil and biochar. Glyphosate usually binds strongly to inorganic soil constituents such as clay particles, iron and aluminium oxides (Mamy and Barriuso 2005; Pessagno et al. 2008; Vereecken 2005). In contrast, diuron is mostly adsorbed to organic matter in the soil (Ahangar et al. 2008) and has been shown by several authors to adsorb strongly to biochar (Martin et al. 2012; Yang et al. 2006; Yang and Sheng 2003).

Based on previous results (Cederlund et al. 2016), our starting hypothesis was that biochar amendment increases diuron adsorption but decreases glyphosate adsorption in soil. The low bioavailability of diuron should lead to slower degradation rates, while the higher bioavailability of glyphosate should lead to it being degraded faster. To test this hypothesis, the behaviour of the two herbicides was compared in one clayey and one sandy arable soil which were amended with biochar. To estimate the effect of ageing, soil-biochar mixtures were incubated at 20 °C for 3.5 months, then adsorption and degradation of the two herbicides were measured. In addition, their fate was studied in a unique, historically charcoal-enriched soil that had been amended with charred organic matter from charcoal kilns for 150 years until the 1950s.

## Materials and Methods

### Soil Sampling and Processing

The soil samples were collected in September 2015 from arable fields at two locations: Länna (L) (59° 52′ N, 17° 58′ E) and Ulleråker (U) (59° 49′ N, 17° 39′ E). Soil sampling at L was performed, according to the scheme shown in Supplementary Fig. S1, in two parts of the arable field: an untreated part (L) and a historically charcoal-enriched part (LB). Because of the long-term charcoal amendment, the latter soil was characterised by lower bulk density and higher loss on ignition and water-holding capacity (WHC) than the unamended soil from the same field, which leads to higher yields in dry years (Tor Kihlberg et al. unpublished). In each soil, about 10 samples were taken from the upper layer (5–15 cm below surface) and pooled. After sieving, the Ø < 2 mm fraction was homogenised and stored at −20 °C in plastic bags until the start of the experiment. Moisture content and WHC were measured for all soil samples. Moisture content was determined by drying at 110 °C for 10 h, while WHC was defined as the moisture content after saturation of 30 g soil with distilled water for 10 h followed by 4 h of free drainage. Chemical and physical properties of the three soils studied (L, LB, U) were determined by a commercial laboratory and are presented in Tables 1 and 2.

**Table 1:** Chemical properties of the soils studied

Soil	Code	HCl extracted K (mg 100 g^−1^)	HCl extracted P (mg 100 g^−1^)	Al-K^a^ (mg 100 g^−1^)	Al-P^a^ (mg 100 g^−1^)	Total C (%)	Total N (%)	pH
Charcoal-amended soil from Länna	LB	68.35	85.02	3.82	16.48	17.57	0.37	5.57
Untreated soil from Länna	L	229.36	78.77	37.28	16.67	4.86	0.34	5.27
Soil from Ulleråker	U	287.59	68.16	34.95	4.87	1.36	0.1	6.41

^a^Al–K/Al-P = ammonium lactate-extractable K and P—Swedish standard method for estimation of plant available K and P fractions (Otabbong et al. 2009)

**Table 2:** Physical properties of the soils studied

Soil code	Clay Ø < 0.002 mm	Fine silt 0.002–0.006 mm	Medium silt 0.006–0.02 mm	Coarse silt 0.02–0.06 mm	Fine sand 0.06–0.2 mm	Medium sand 0.2–0.6 mm	Coarse sand 0.6–2 mm	Loss on ignition %
LB	n.d.	n.d.	n.d.	n.d.	5.9	3.3	4.1	39.4
L	66.5	14.8	9.1	6.5	2.1	0.7	0.3	13.7
U	7.5	3.2	2.4	3.2	12.1	63.8	7.8	3.3

*n.d.* not determined

### Preparation and Ageing of Soil-Biochar Mixtures

The biochar used was the commercial product *Skogens kol*, which is produced from a mixture of about 80% hardwood, mainly birchwood (*Betula* sp*.*) and 20% wood from Norway spruce (*Picea abies*), by slow pyrolysis with a maximum process temperature of 380–430 °C (Cederlund et al. 2016). A sample of the biochar was sent to Eurofins for determination of some physicochemical properties and the results are presented in Table 3.

**Table 3:** Some physicochemical properties of the biochar “Skogens kol” used in this study

BET-specific surface area (m^2^/g)	True density (g/cm^3^)	pH (H_2_O/CaCl_2_)	Ash content 550 °C (% w/dw)	Nitrogen (% w/dw)	Carbon (% w/dw)	Hydrogen (% w/dw)	Oxygen (% w/dw)	H/C ratio (molar)	O/C ratio (molar)	Volatile compounds (% w/dw)
161.3	1.48	8.4/7.9	9.8	0.57	83.5	2.7	4.1	0.38	0.037	12.8

Soil-biochar mixtures were prepared by mixing soil (L and U) with sieved biochar (Ø < 2 mm) at a rate of 1, 10, 20 and 30% biochar per unit soil dry weight (designated L1, L10, L20 and L30 and U1, U10, U20 and U30). WHC was determined as described above and pH for all mixtures was measured in a 1:2 slurry of soil and distilled water (*w*/*v*) after shaking and stabilisation for 10 h (Table 4).

**Table 4:** Moisture content, water-holding capacity (WHC) and pH of soil samples and soil-biochar mixtures

Soil/mixture code^a^	WHC (%)	pH
LB	57	5.77
L	53	5.27
L1	51	5.3
L10	51	5.55
L20	51	5.75
L30	51	6.07
U	27	6.41
U1	29	6.53
U10	35	6.88
U20	38	7.15
U30	42	7.4
U1a^b^	Not measured	6.45
U10a		6.94
U20a		7.45
U30a		7.69

^a^Mixtures named by soil from which they were made and biochar percentage

^b^a denotes aged mixtures

Biochar ageing was performed with soil-biochar mixtures made from U soil. These mixtures were incubated in darkness at 20 °C for 3.5 months. The moisture content was adjusted to 55% of WHC and monitored and adjusted weekly by addition of deionised water.

### Chemicals Used in Herbicide Adsorption and Degradation Experiments

Glyphosate (N-(phosphonomethyl)-glycine, CAS [1071-83-6], 98%) and diuron (3-(3,4-dichlorophenyl)-1,1-dimethylurea (DCMU), CAS [330-54-1], 99.0%) were provided by Dr. Ehrenstorfer GmbH, Augsburg, Germany. Their chemical properties are presented in Supplementary Table 1. ^14^C-labelled diuron ([ring-U-14C], 96.4%, 5.71 MBq mg^−1^) and glyphosate ([P-methylene-14C], 4.87 MBq mg^−1^) were provided by the Institute of Isotopes Co. Ltd., Budapest, Hungary.

### Measurement of Herbicide Adsorption in Soils and Soil-Biochar Mixtures

Adsorption was determined in a batch-equilibrium system according to OECD guideline 106 (OECD 2000). A pre-study was performed to estimate the time when the equilibrium between adsorbed herbicide and herbicide in solution was reached (8, 24 and 32 h). In all cases, equilibrium was reached within 24 h. For high-percentage soil-biochar mixtures with U soil, an additional pre-study was performed to estimate an appropriate soil to solution ratio as defined in the OECD guideline.

Soil and soil-biochar mixtures, corresponding to 1 g of soil or mixture dry weight, were weighed into tubes (15-mL glass tubes for diuron and 50-mL polypropylene tubes for glyphosate) and adjusted with 0.01 M CaCl_2_ to reach the appropriate soil-solution ratio. This was 1:40 for all samples with glyphosate and for U20, U30, U20a and U30a with diuron and 1:4 for all other samples with diuron. The samples were shaken for 24 h (20 °C, 200 revolutions min^−1^). After that, herbicides were added to reach concentrations of 1, 5, 10, 50 and 100 μg g^−1^ dry weight (dw) soil for glyphosate and 0.1, 0.5, 1, 5 and 10 μg g^−1^ dw soil for diuron, due to its lower water solubility. In addition, a fixed amount (10 μL for glyphosate and 20 μL for diuron) of ^14^C-labelled herbicide was added to each tube to reach an activity of 2000 DPM (3.333 × 10^−5^ MBq) per sample. There were two replicate tubes of each concentration. After 24 h, the tubes were centrifuged (3000 revolutions min^−1^ for 30 min), samples of supernatant were transferred to scintillation vials (4 mL for diuron and 10 mL for glyphosate samples) and Quicksafe A (Scintvaruhuset, LAB-service, Uppsala, Sweden) was added directly before measurement of scintillation. ^14^C activity was measured on a Beckman LS 6000TA liquid scintillation counter (Beckman Counter Inc., Fullerton, CA). Controls without herbicides were measured for all samples to exclude the level of background radioactivity. The data obtained were fitted using the linear form of the Freundlich equation:

$$ \log {q}_e= \log {K}_f+ n \log {C}_e $$

where *q*_*e*_ (μg g^−1^) is the adsorbed amount, *C*_*e*_ (μg mL^−1^) is the equilibrium concentration in the aqueous phase, *K*_*F*_ (μg^1–1/*n*^ g^−1^ mL^1/*n*^) is the Freundlich adsorption coefficient and *n* is the Freundlich exponent.

### Herbicide Degradation Experiment

The herbicides were dissolved in water (glyphosate) or methanol (diuron) and added dropwise to a fraction (10%) of the soils and soil-biochar mixtures. Water and methanol were allowed to evaporate from the samples for 10 h. The herbicide-treated part was then mixed with the rest of each sample to give an initial nominal concentration of 10 mg kg^−1^ soil dry weight. Portions of soil corresponding to 5 g of dry weight were weighed into 50-mL plastic tubes and the water content was adjusted to 60% of WHC and kept at this level for the duration of the experiment. The tubes were sealed with caps and were incubated at 20 °C in the dark. After 1, 2, 5, 8, 16, 23 (only for U samples) and 31 days of incubation, two replicate tubes from each treatment were placed in the freezer (−20 °C) for future extraction and analysis.

Data from the degradation experiment after recovery correction were used to estimate herbicide half-life. Recovery was calculated as:

$$ \mathrm{Recovery}=\left(\frac{C_0}{C_{\mathrm{nominal}}}\right)\times 100 $$

where *C*_*0*_ is the herbicide concentration determined at day 0.

Natural logarithms of remaining concentrations for days 0–31 were plotted against time, giving the first-order rate constant *k* as the slope of the linear regression line. Half-life (*T*½) was calculated as:

$$ T\frac{1}{2}=\frac{ \ln 2}{k} $$

### Analysis of Diuron

For diuron extraction from soil and soil-biochar mixtures, the following protocol was used: 10 mL methanol were added using a Vogel pipette to the tubes with sample. The tubes were shaken at 200 revolutions min^−1^ for 60 min, centrifuged at 4000 revolutions min^−1^ for 10 min and the supernatant was filtered (OOH Whatman; 11 cm). Portions (1 mL) of filtrate were transferred to sample vials and HPLC analysis was performed according to the protocol in Cederlund et al. (2007). Standard solutions with concentration range 0.05–50 μg mL^−1^ were analysed with extracts from samples. The HPLC was equipped with a G1314A UV detector, a G1311A pump, a G1329A auto injector (Agilent Technologies AB; 1100 Series; Sweden) and a Zorbax SB-C18 column (12.5 × 4.6 mm, 5 mm; ChromTech AB, Sundbyberg, Sweden).

### Analysis of Glyphosate

Extraction of glyphosate, derivatisation and measurement on GC-MS were performed using the same reagents for analytical standards, glyphosate extraction and internal standards as previously described (Bergström, Börjesson, and Stenström, 2011). The instrument was a Hewlett-Packard 6890 GC (Agilent Technologies Sweden AB), equipped with a 30 m by 0.25-mm diameter fused silica capillary column with 0.25-μm film thickness (HP-5 for GC-MS), a mass spectrometer 5973, a split/splitless injector and the software Chemstation (Agilent Technologies, Kista, Sweden).

## Results

### Effect of Biochar on Soil Water-Holding Capacity and pH

**Fig. 1:**
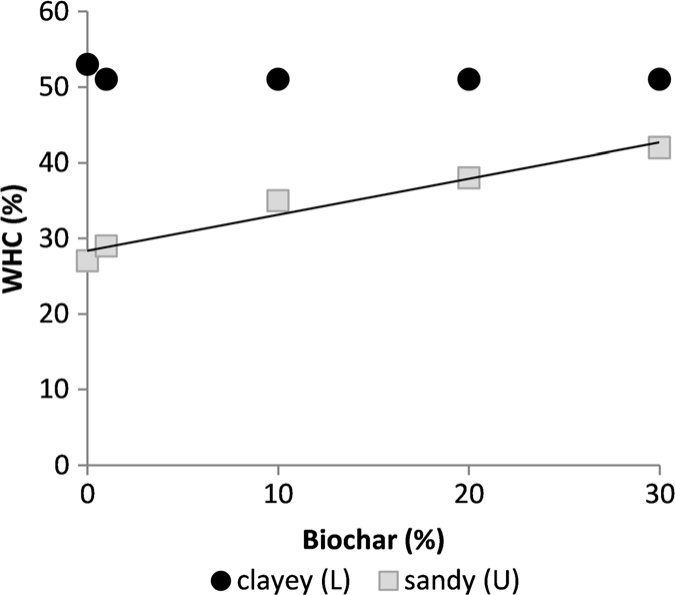
Water-holding capacity (WHC) of the soil samples ± standard deviations plotted against biochar percentage added

The studied soils had different physical texture: the dominant particle fractions in the L soil were clay and fine silt, while the U soil was dominated by medium and fine sand. The texture of the LB soil could not be fully determined due to its high organic matter content, as traces of organic C remained in the sample after digestion (oxidation by H_2_O_2_). Coming from the same field as L, it is likely that the LB soil was also dominated by clay. However, the proportion of sand was higher (Table 2). This agrees with Kihlberg et al. (unpublished), who also reported a coarser particle size distribution in LB compared with L soil, but also did not subdivide particles with Ø < 0.06 mm. The WHC of the clayey L soil (53%) was higher than in the sandy U soil, where it was only 27%, and was not affected by biochar addition. However, the LB soil, which was historically amended by charcoal, had a higher WHC (57%) than the L soil with or without fresh biochar amendment. In the sandy soil, the WHC increased from 27 to 42% with biochar addition and was correlated positively (*r* = 0.98) with the biochar percentage (Fig. 1).

**Fig. 2:**
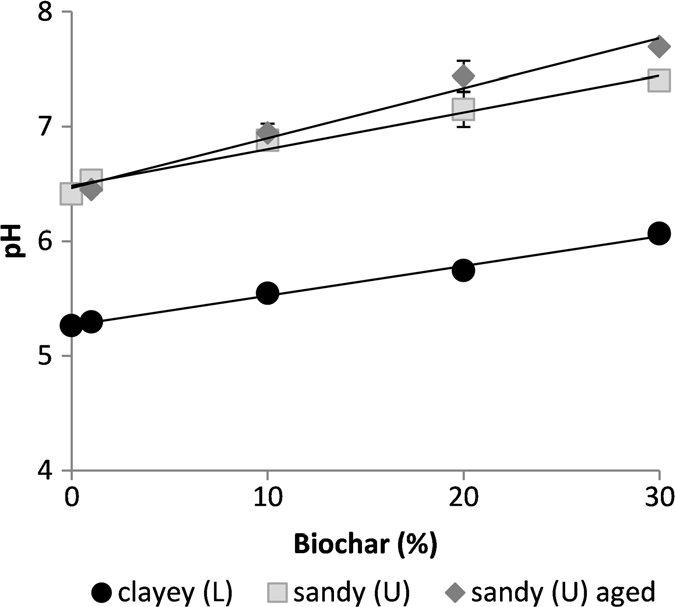
pH of the soil samples (N = 2) ± standard deviations plotted against biochar percentage added

Biochar addition increased the pH from 5.27 to 6.07 in the L soil and from 6.41 to 7.69 in the U soil (Table 4; Fig. 2). Ageing of the biochar led to a further pH increase in most of the soil-biochar mixtures (U10a-U30a). In the LB soil, the pH was higher (5.77) than in the L soil. The pH of soil-biochar mixtures was correlated with the percentage of biochar added in all cases (*r* = 0.99 for L soil-biochar mixtures; *r* = 0.99 for fresh U soil-biochar mixtures; *r* = 0.98 for aged U soil-biochar mixtures).

### Adsorption of Diuron

**Fig. 3:**
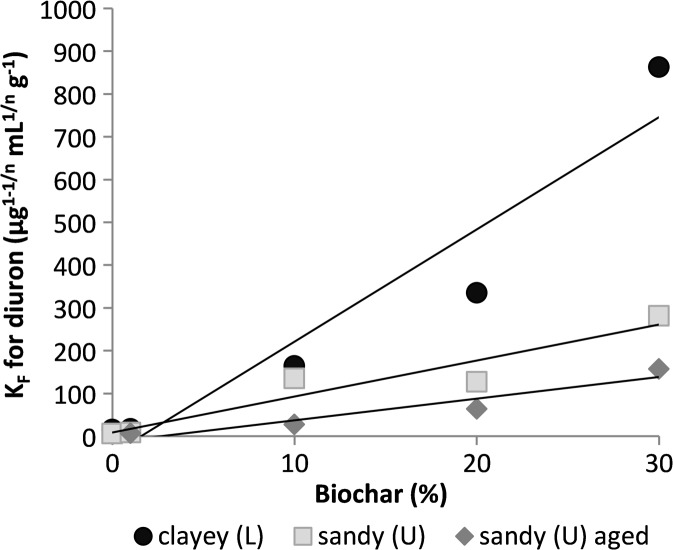
Freundlich *K*_*F*_ values for diuron plotted against biochar percentage added in samples from Länna (L) and Ulleråker (U)

Biochar amendment increased diuron adsorption in both the L and U soils (Fig. 3). In the LB soil, *K*_*F*_ was 364 μg^1–1/*n*^(mL) ^1/*n*^ g^−1^, which is quite close to the *K*_*F*_ value of the L20 soil-biochar mixture. *K*_*F*_ values in the aged soil-biochar mixtures were lower than in mixtures with fresh biochar addition. There were positive correlations between the diuron *K*_*F*_ values and biochar percentage for L, U and aged U soils (*r* = 0.96, *r* = 0.95, and *r* = 0.95, respectively).

### Adsorption of Glyphosate

**Fig. 4:**
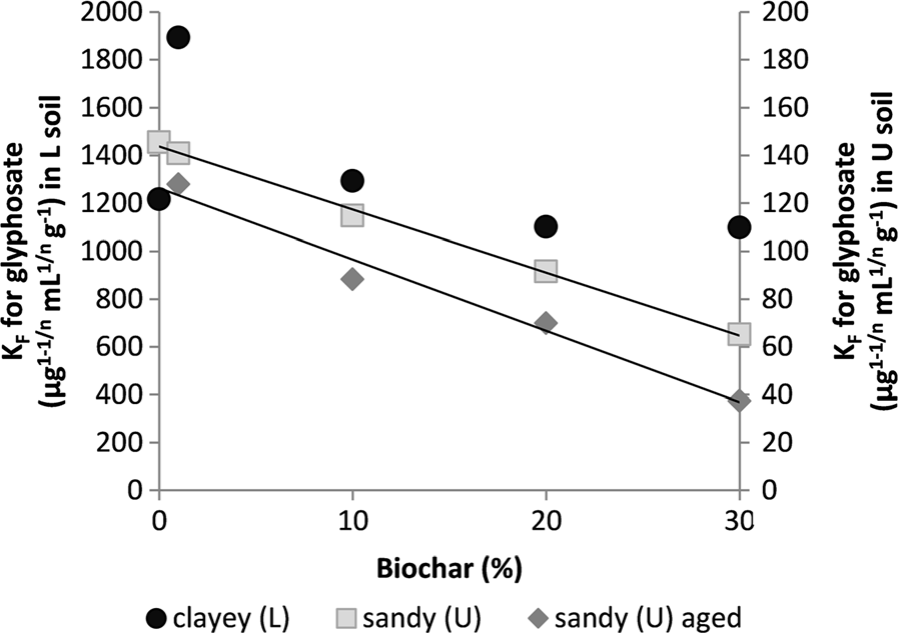
Freundlich *K*_*F*_ values for glyphosate plotted against biochar percentage added in samples from Länna (L) and Ulleråker (U)

Glyphosate was more strongly adsorbed in the L soil (*K*_*F*_ = 1218 μg^1–1/*n*^ mL^1/*n*^ g^−1^) than in the U soil (*K*_*F*_ = 146 μg^1–1/*n*^ mL^1/*n*^ g^−1^). No consistent effect of biochar amendment on glyphosate adsorption in L soil was observed (Fig. 4). A very high *K*_*F*_ value was observed for the sample with 1% biochar addition (*K*_*F*_ = 1892 μg^1–1/*n*^ mL^1/*n*^ g^−1^), while the *K*_*F*_ values for the unamended L soil and the other soil-biochar mixtures varied between 1099 and 1294 μg^1–1/*n*^ mL^1/*n*^ g^−1^. The LB soil had a much lower *K*_*F*_ value (539 μg^1–1/*n*^ mL^1/*n*^ g^−1^) than the L soil and soil-biochar mixtures. However, in the U soil, glyphosate adsorption was correlated negatively (*r* = −0.99) with the biochar percentage (Fig. 4). Ageing of the biochar decreased adsorption further.

### Degradation of Diuron

**Fig. 5:**
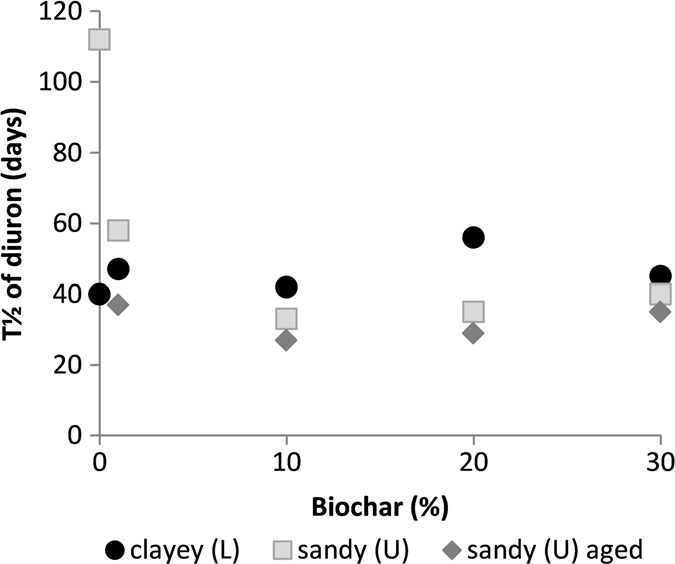
Diuron half-life in the Länna (L) and Ulleråker (U) soils and soil-biochar mixtures

In the L and U soils and soil-biochar mixtures, from 20 to 50% of the added diuron was degraded during the experimental period. Diuron half-life varied between 40 to 56 days in the L soil, was 36 days in the LB soil and varied between 26 to 112 days in the U soil (Fig. 5). No correlation was seen between the biochar percentage and diuron half-life in any of the soils. However, in the U soil, the half-life was shorter in all samples with biochar addition compared with the unamended soil. Here, it should be noted that the half-life of 112 days found for the U soil without biochar may be a less accurate estimation, since the dynamics of diuron degradation did not fit well with a first-order kinetic model in this sample. The degradation kinetics of all other samples followed first-order kinetics reasonably well, with *R* ^2^ values of 0.7–0.96. Ageing of the biochar consistently decreased diuron half-life in the U soil.

### Degradation of Glyphosate

In the L and U soils and soil-biochar mixtures, 10–70% of the added glyphosate was degraded during the experimental period. Glyphosate half-life in the L soil varied between 51 and 187 days. However, in the L, L1, L10 and L20 samples, the data fitted poorly to the first-order kinetic model (*R* ^2^ = 0.33–0.61), mostly due to great variation in glyphosate concentrations during the first week of degradation. This fact can explain the somewhat inconsistent pattern of half-life variation for the soil-biochar mixes. However, degradation in the LB and L30 samples followed first-order kinetics well (*R* ^2^ = 0.97 and 0.94). The shortest glyphosate half-life (19 days) was observed in the LB soil.

**Fig. 6:**
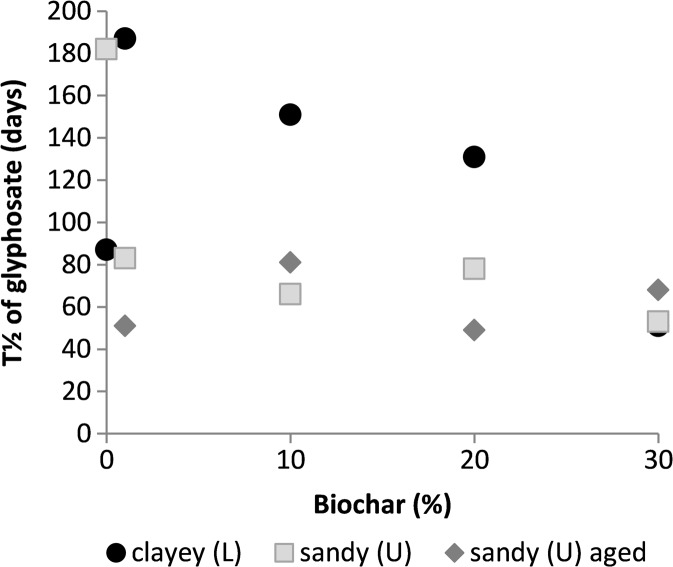
Glyphosate half-life in the Länna (L) and Ulleråker (U) soils and soil-biochar mixtures

Degradation of glyphosate was relatively slow in the unamended U soil, but was faster in all samples with biochar amendment. In the unamended U soil, the half-life of glyphosate was 182 days, while in the U soil-biochar mixtures, it varied between 49 and 83 days. However, as in the case of diuron, data from the unamended U soil were a poor fit to the first-order model (*R* ^2^ = 0.48) and the degradation rate in the biochar-amended samples did not appear to be related to the biochar percentage added. The fastest degradation was observed in the U1a and U20a soil-biochar mixtures, but ageing of the biochar did not consistently affect degradation rates (Fig. 6).

**Fig. 7:**
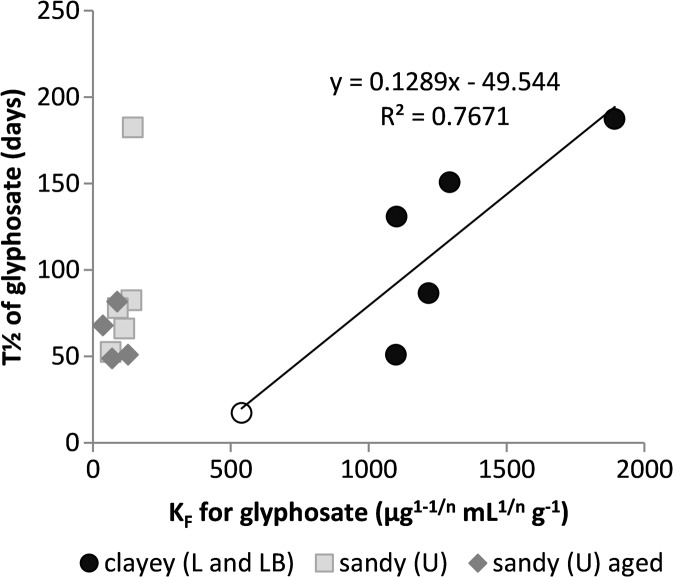
Correlation between glyphosate half-life and adsorption coefficient (*K*_*F*_). The open circle is for the LB soil

No correlations between glyphosate half-life and amount of added biochar were found for any of the L and U soils (Fig. 6). However, the half-life was correlated with the *K*_*F*_ value for glyphosate (*r* = 0.88) in samples of the L soil when the LB sample was included (Fig. 7). In the U soil and soil-biochar mixtures, the adsorption coefficient of glyphosate was generally lower and its half-life was not correlated with the *K*_*F*_ value (Fig. 7).

## Discussion

### Biochar Influence on Soil Properties

Biochar is well-known for its ability to increase water retention in soils (Basso et al. 2013; Yu et al. 2013). According to a recent meta-analysis, biochar amendment increases the available WHC by 15% on average, but a significant effect is only seen in coarse textured-soils (Omondi et al. 2016). This is supported by our observation that biochar addition only increased WHC in the sandy U soil and not in the clayey L soil. Water uptake by biochar is mainly regulated by its porosity and hydrophobicity (Gray et al. 2014). Thus, considering the low wettability but high porosity of the biochar that we studied (Cederlund et al. 2016), it is likely that the increase in WHC in the sandy soil was mediated by an increase in overall soil porosity. We did not determine the WHC after the short-term ageing experiment. However, the higher WHC in the LB soil in comparison with L soil may indicate that the long-term ageing of the charcoal in that soil had increased its water retention capacity. Oxidation of biochar during prolonged environmental exposure can introduce oxygen-containing groups on its surface (Sorrenti et al. 2016) and these would increase water uptake by the biochar (Suliman et al. 2017).

Biochar mediated a pH increase in both soils that was proportional to the percentage of added biochar. Similarly, a meta-analysis by Biederman and Harpole (2013) found that biochar generally increases soil pH, especially in acidic soils. The increase in pH during the process of ageing can be explained by release of basic salts from the biochar (Joseph et al. 2010).

### Effects of Biochar on Herbicide Adsorption

Diuron adsorption increased after biochar amendment in both the L and U soils. This effect of biochar addition has been observed in previous studies for silty loam (Yang et al. 2006) and sandy soil (Yu et al. 2006). Biochar contains many adsorption sites that can bind non-polar herbicides, so diuron adsorption increased with amount of biochar added, and the risk of it leaching is lower (Kookana 2010). The increased pH obtained with biochar addition is not likely to have contributed to the increased sorption since diuron is uncharged at relevant soil pH-levels. In a previous study, we also found that pH has no effect on diuron adsorption when studying this particular biochar without soil (Cederlund et al. 2016).

Biochar addition decreased glyphosate adsorption in the sandy U soil, but not in the clayey L soil. The difference in effects of biochar on glyphosate adsorption between the L and U soils may be explained by the different soil texture and physical properties of these soils. The decreased glyphosate adsorption in the U soil is likely to be related to the induced pH changes. According to several studies, soil pH is negatively correlated with glyphosate adsorption (Gimsing et al. 2004b; Mamy and Barriuso 2005; Vereecken 2005). Increased soil pH can increase the negative charge of both soil surfaces and glyphosate itself, which leads to enhanced repulsion. Glyphosate has a pH-dependent OH− group with a pKa value of 5.7, so its charge is likely to have been affected in the pH range studied here. The same relationship with pH has been observed for glyphosate adsorption on pure biochar: Herath et al. (2016) studied the effect of pH on adsorption of glyphosate on a rice husk biochar and found that the adsorption percentage varied from 75 to 85% at pH 3–5, decreased to 75–65% at pH 6–8 and then significantly dropped to 55% at pH 9. However, in a previous study, we showed that glyphosate adsorption by the studied biochar was low at both low and high pH (Cederlund et al. 2016).

In the L soil, there was no linear relationship between glyphosate adsorption coefficient and biochar amendment. The overall strong adsorption in this soil possibly contributed to masking the relatively minor effects of the biochar. It is known that inorganic components of soil, such as Al- and Fe-oxides, adsorb glyphosate effectively (Gimsing et al. 2004a) and that this herbicide is less available in soils with a high clay content. The induced pH changes in this soil also occurred over a different pH interval, which may have contributed to the less clear outcome.

### Effects of Biochar Ageing on Adsorption

Short-term ageing of the biochar mixtures in the laboratory decreased adsorption of both herbicides. This suggests that processes that have the potential to reduce sorption, such as organo-mineral interactions with the biochar surface (Pignatello et al. 2006; Singh and Kookana 2009; Lin et al. 2012), were the dominant forces affecting the biochar during our ageing experiment. For diuron, our results are consistent with findings in a field study on biochar amendment of Australian ferrosols, in which diuron and atrazine adsorption to soils amended by poultry litter and paper mill biochar was significantly reduced after 32 months of ageing (Martin et al. 2012). For glyphosate, it is possible that the further increase in pH during the 3 months of ageing contributed to the additional decrease observed in sorption. Although we cannot know the original properties of the charcoal applied to the historically charcoal-enriched LB soil, it may be informative to compare the adsorption results from this soil. In LB, the *K*_*F*_ value for diuron was comparable to that determined in the 20% soil-biochar mixture (L20) and, considering that the total carbon content of the LB soil is about 18% (Table 1), this suggests limited effects of ageing. However, for glyphosate, the *K*_*F*_ value of the LB soil was only 539 μg^1–1/*n*^ mL^1/*n*^ g^−1^, which is only about half the *K*_*F*_ value found for any of the fresh biochar mixtures or the unamended L soil (Table 5). Since the adsorption of glyphosate on the biochar itself is very weak, this low adsorption is difficult to explain in terms of reduced adsorptive affinity of the charcoal. It is more likely to reflect a reduced affinity for glyphosate of the soil itself. Kihlberg et al. (unpublished) suggest that the heat from the charcoal kilns in LB may have contributed to sintering the clay particles in the soil, causing a shift towards a coarser particle size distribution. Heating clay soils to 500 °C has been shown to change soil physical texture and increase the amount of silt and sand particles (Badía and Martí 2003). Such a reduction in the proportion of clay would consequently reduce the amount of surfaces available for glyphosate adsorption. Heating may also cause other mineralogical changes in soil that affect adsorption, for instance de Santana et al. (2006) reported reduced interaction between glyphosate and Al_2_O_3_ and Fe_2_O_3_ in soil after burning.

**Table 5:** Freundlich parameters (*K*_*F*_, 1/*n* and *R* ^2^ value) for adsorption and half-life of diuron and glyphosate

Sampling site		Adsorption						Degradation			
		Diuron			Glyphosate			Diuron		Glyphosate	
		*K*_*F*_^a^	1/*n*	*R* ^2^	*K*_*F*_^a^	1/*n*	*R* ^2^	*T*½^b^	*R* ^2^	*T*½^b^	*R* ^2^
Länna	LB	364	0.859	0.99	539	0.890	0.99	36	0.965	17	0.97
	L	15.21	0.863	0.99	1218	0.842	0.99	40	0.963	87	0.606
	L1	17.10	0.807	0.99	1892	0.872	0.99	47	0.708	187	0.333
	L10	164	0.859	0.99	1294	0.806	0.99	42	0.853	151	0.385
	L20	335	0.822	0.99	1102	0.796	0.99	56	0.918	131	0.402
	L30	863	0.978	0.96	1099	0.780	0.98	45	0.86	51	0.945
Ulleråker	U	5.73	0.798	0.99	145.5	0.783	0.99	112	0.663	182	0.482
	U1	8.60	0.586	0.95	140.9	0.765	0.99	58	0.718	83	0.767
	U10	135	0.789	0.99	114.8	0.754	0.99	33	0.866	66	0.674
	U20	127	0.727	0.85	91.6	0.780	0.99	35	0.868	78	0.621
	U30	281	0.753	0.97	65.2	0.750	0.99	40	0.888	53	0.861
	U1a	6.44	0.760	0.99	127.9	0.761	0.99	37	0.71	51	0.716
	U10a	27.4	0.824	0.99	88.3	0.776	0.99	27	0.785	81	0.683
	U20a	64	0.547	0.94	70.0	0.788	0.99	29	0.849	49	0.917
	U30a	157	0.686	0.97	37.4	0.751	0.99	35	0.871	68	0.885

^a^The unit of *K*_*F*_ is μg^1–1/*n*^ mL^1/*n*^ g^−1^

^b^The unit of *T*½ is days

Our results for glyphosate differ somewhat from those of Kumari et al. (2016), who found that glyphosate sorption was increased in a silty loam soil amended with the same wood-based biochar that we used (*Skogens kol*) after 7–10 months of ageing under field conditions. The application rates used in their study varied from 10 to 100 Mg biochar ha^−1^ added to the topsoil layer (0–10 cm), which corresponds to about 0.8–8% of biochar per gramme dry weight assuming a bulk density of the soil of 1.3 g cm^−3^. Increases in glyphosate sorption occurred in plots amended with 10, 20 and 40 Mg ha^−1^ of biochar (i.e. corresponding to 0.8, 1.6 and 3.2% *w*/*w*), while the plot amended with 100 Mg ha^−1^, where the glyphosate adsorption was the same as in the unamended plots, was considered to be an outlier (Kumari et al. 2016). In the present study, the clayey L soil with the lowest application rate was the outlier: the adsorption coefficient in the L1 soil-biochar mixture was much higher than in L soil without amendment, while the adsorption coefficient in the L10, L20 and L30 soil-biochar mixtures was the same or lower than in the unamended clayey L soil. However, we cannot offer an explanation for this pattern. In the sandy U soil, the adsorption of glyphosate was reduced after the ageing process, which can be explained by a further pH increase and low affinity to sorb glyphosate in both sandy soil and biochar itself.

### Herbicide Degradation before and after Biochar Amendment

Microbial degradation of chemicals in soil has often been reported to be limited by strong sorption (Bergström et al. 2011; Gimsing et al. 2004a; Wu et al. 2011). Moreover, pesticide degradation is often inhibited after fresh biochar addition (Kookana 2010), which can be explained by a decrease in their bioavailability. In the present case, it seems that despite the fact that adsorption of diuron increased in both soils and that adsorption of glyphosate decreased in the sandy soil, biochar amendment had no clear effect on either diuron or glyphosate degradation. However, even though neither the *K*_*F*_ value nor the half-life of glyphosate was clearly correlated with the added biochar percentage in the clayey L soil, the half-life was correlated with the *K*_*F*_ value (Fig. 7). This indicates that in the case of glyphosate in the clayey L soil, which had *K*_*F*_ values >1000 μg^1–1/*n*^ mL^1/*n*^ g^−1^, availability of glyphosate may have been a rate-limiting factor for its degradation, while in the other cases adsorption was too weak to have an effect.

### Conclusions

As hypothesised, fresh biochar addition increased diuron adsorption in both clayey (L) and sandy (U) soils. However, glyphosate adsorption decreased only in the sandy U soil. These effects are most likely due to adsorption of diuron on the biochar itself, while in the case of glyphosate the decreased sorption may be explained by an increase in soil pH after biochar addition. No consistent effect of biochar amendment on herbicide degradation was observed in the studied soils, which contradicts our initial hypothesis. However, there was a positive relationship between adsorption and glyphosate half-life in the clayey soil-biochar mixtures, indicating that availability may be the rate-limiting step, but only where adsorption is strong. The consequences of biochar ageing under laboratory conditions were further increases in soil pH and a reduction in adsorption of both herbicides. Changes in biochar adsorptive properties during ageing in soil should be taken into consideration when planning its use in agriculture and for soil remediation purposes.

## Electronic supplementary material

Fig. S1 (DOCX 114 kb)

Supplementary Table 1 (DOCX 14 kb)

## References

[CR1] Ahangar AG, Smernik RJ, Kookana RS, Chittleborough DJ (2008). Clear effects of soil organic matter chemistry, as determined by NMR spectroscopy, on the sorption of diuron. Chemosphere.

[CR2] Ahmad M, Rajapaksha AU, Lim JE, Zhang M, Bolan N, Mohan D (2014). Biochar as a sorbent for contaminant management in soil and water: a review. Chemosphere.

[CR3] Badía D, Martí C (2003). Plant ash and heat intensity effects on chemical and physical properties of two contrasting soils. Arid Land Research and Management.

[CR4] Basso AS, Miguez FE, Laird DA, Horton R, Westgate M (2013). Assessing potential of biochar for increasing water-holding capacity of sandy soils. GCB Bioenergy.

[CR5] Beesley L, Moreno-Jiménez E, Gomez-Eyles JL, Harris E, Robinson B, Sizmur T (2011). A review of biochars’ potential role in the remediation, revegetation and restoration of contaminated soils. Environmental Pollution.

[CR6] Bergström L, Börjesson E, Stenström J (2011). Laboratory and lysimeter studies of glyphosate and aminomethylphosphonic acid in a sand and a clay soil. Journal of Environmental Quality.

[CR7] Biederman LA, Harpole S (2013). Biochar and its effects on plant productivity and nutrient cycling: a meta-analysis. Global Change Biology Bioenergy.

[CR8] Cayuela ML, van Zwieten L, Singh BP, Jeffery S, Roig A, Sánchez-Monedero MA (2014). Biochar’s role in mitigating soil nitrous oxide emissions: a review and meta-analysis. Agriculture, Ecosystems & Environment.

[CR9] Cederlund H, Börjesson E, Lundberg D, Stenström J (2016). Adsorption of pesticides with different chemical properties to a wood biochar treated with heat and iron. Water Air and Soil Pollution.

[CR10] Cederlund H, Börjesson E, Önneby K, Stenström J (2007). Metabolic and cometabolic degradation of herbicides in the fine material of railway ballast. Soil Biology & Biochemistry.

[CR11] Cheng CH, Lehmann J, Engelhard MH (2008). Natural oxidation of black carbon in soils: changes in molecular form and surface charge along a climosequence. Geochimica et Cosmochimica Acta.

[CR12] de Santana, H., Toni, L. R. M., Benetoli, L. O. de B., Zaia, C. T. B. V., Rosa, M., & Zaia, D. A. M. (2006). Effect in glyphosate adsorption on clays and soils heated and characterization by FT-IR spectroscopy. *Geoderma, 136*(3), 738–750. doi:10.1016/j.geoderma.2006.05.012.

[CR13] Gimsing AL, Borggaard OK, Bang M (2004). Influence of soil composition on adsorption of glyphosate and phosphate by contrasting Danish surface soils. European Journal of Soil Science.

[CR14] Gimsing AL, Borggaard OK, Jacobsen OS, Aamand J, Sørensen J (2004). Chemical and microbiological soil characteristics controlling glyphosate mineralisation in Danish surface soils. Applied Soil Ecology.

[CR15] Gray, M., Johnson, M. G., Dragila, M. I., & Kleber, M. (2014). Water uptake in biochars: The roles of porosity and hydrophobicity. *Biomass and Bioenergy, 61*, 196–205. doi:10.1016/j.biombioe.2013.12.010.

[CR16] Hale, S. E., Hanley, K., Lehmann, J., Zimmerman, A. R., & Cornelissen, G. (2011). Effects of chemical, biological, and physical aging as well as soil addition on the sorption of pyrene to activated carbon and biochar. *Environmental Science & Technology, 45*(24), 10445–10453. doi:10.1021/es202970x. 10.1021/es202970x 22077986

[CR17] Herath I, Kumarathilaka P, Al-Wabel MI, Abduljabbar A, Ahmad M, Usman ARA, Vithanage M (2016). Mechanistic modeling of glyphosate interaction with rice husk derived engineered biochar. Microporous and Mesoporous Materials.

[CR18] Jablonowski ND, Borchard N, Zajkoska P, Fernández-Bayo JD, Martinazzo R, Berns AE, Burauel P (2013). Biochar-mediated [14C]atrazine mineralization in atrazine-adapted soils from Belgium and Brazil. Journal of Agricultural and Food Chemistry.

[CR19] Jeffery S, Verheijen FGA, vad der Velde M, Bastos AC (2011). A quantitative review of the effects of biochar application to soils on crop productivity using meta-analysis. Agriculture, Ecosystems and Environment.

[CR20] Jones DL, Edwards-Jones G, Murphy DV (2011). Biochar mediated alterations in herbicide breakdown and leaching in soil. Soil Biology and Biochemistry.

[CR21] Joseph SD, Camps-Arbestain M, Lin Y, Munroe P, Chia CH, Hook J (2010). An investigation into the reactions of biochar in soil. Australian Journal of Soil Research.

[CR22] Kookana RS (2010). The role of biochar in modifying the environmental fate, bioavailability, and efficacy of pesticides in soils: a review. Australian Journal of Soil Research.

[CR23] Kumari KGID, Moldrup P, Paradelo M, Elsgaard L, de Jonge LW (2016). Soil properties control glyphosate sorption in soils amended with birch wood biochar. Water Air and Soil Pollution.

[CR24] Lin Y, Munroe P, Joseph S, Kimber S, Van Zwieten L (2012). Nanoscale organo-mineral reactions of biochars in ferrosol: an investigation using microscopy. Plant and Soil.

[CR25] Mamy L, Barriuso E (2005). Glyphosate adsorption in soils compared to herbicides replaced with the introduction of glyphosate resistant crops. Chemosphere.

[CR26] Martin SM, Kookana RS, Van Zwieten L, Krull E (2012). Marked changes in herbicide sorption-desorption upon ageing of biochars in soil. Journal of Hazardous Materials.

[CR27] OECD. (2000). Adsorption-desorption using a batch equilibrium method. *Oecd Guideline for the Testing of Chemicals*, (January), pp. 1–44. doi:10.1787/9789264069602-en.

[CR28] Omondi MO, Xia X, Nahayo A, Liu X, Korai PK, Pan G (2016). Quantification of biochar effects on soil hydrological properties using meta-analysis of literature data. Geoderma.

[CR29] Otabbong E, Börling K, Kätterer T, Mattsson L (2009). Compatibility of the ammonium lactate (AL) and sodium bicarbonate (Olsen) methods for determining available phosphorus in Swedish soils. Acta Agriculturae Scandinavica Section B Soil and Plant Science.

[CR30] Pessagno RC, Torres Sánchez RM, dos Santos Afonso M (2008). Glyphosate behavior at soil and mineral-water interfaces. Environmental Pollution.

[CR31] Pignatello, J. J., Kwon, S., & Lu, Y. (2006). Effect of natural organic substances on the surface and adsorptive properties of environmental black carbon (char): Attenuation of surface activity by humic and fulvic acids. *Environmental Science & Technology, 40*(24), 7757–7763. doi:10.1021/es061307m. 10.1021/es061307m 17256524

[CR32] Safaei Khorram M, Zhang Q, Lin D, Zheng Y, Fang H, Yu Y (2016). Biochar: a review of its impact on pesticide behavior in soil environments and its potential applications. Journal of Environmental Sciences.

[CR33] Sheng G, Yang Y, Huang M, Yang K (2005). Influence of pH on pesticide sorption by soil containing wheat residue-derived char. Environmental Pollution.

[CR34] Singh, N., & Kookana, R. S. (2009). Organo-mineral interactions mask the true sorption potential of biochars in soils. *Journal of Environmental Science and Health, Part B 44*(3), 214–219. doi:10.1080/03601230902728112. 10.1080/03601230902728112 19280473

[CR35] Sorrenti, G., Masiello, C. A., Dugan, B., & Toselli, M. (2016). Biochar physico-chemical properties as affected by environmental exposure. *Science of The Total Environment, 563–564*, 237-246. doi:10.1016/j.scitotenv.2016.03.245. 10.1016/j.scitotenv.2016.03.245 27135586

[CR36] Suliman, W., Harsh, J. B., Abu-Lail, N. I., Fortuna, A-M., Dallmeyer, I., & Garcia-Pérez, M. (2017). The role of biochar porosity and surface functionality in augmenting hydrologic properties of a sandy soil. *Science of The Total Environment, 574*, 139–147. doi:10.1016/j.scitotenv.2016.09.025. 10.1016/j.scitotenv.2016.09.025 27627689

[CR37] Tatarková V, Hiller A, Vaculík M (2013). Impact of wheat straw biochar addition to soil on the sorption, leaching, dissipation of the herbicide (4-chloro-2-methylphenoxy)acetic acid and the growth of sunflower (*Helianthus annuus* L.). Ecotoxicology and Environmental Safety.

[CR38] Trigo C, Spokas KA, Cox L, Koskinen WC (2014). Influence of soil biochar aging on sorption of the herbicides MCPA, nicosulfuron, terbuthylazine, indaziflam, and fluoroethyldiaminotriazine. Journal of Agricultural and Food Chemistry.

[CR39] Vereecken H (2005). Mobility and leaching of glyphosate: a review. Pest Management Science.

[CR40] Wu X, Li M, Long Y, Liu R (2011). Effects of adsorption on degradation and bioavailability of metolachlor in soil. Journal of Soil Science and Plant Nutrition.

[CR41] Yang Y, Sheng G (2003). Pesticide adsorptivity of aged particulate matter arising from crop residue burns. Journal of Agricultural and Food Chemistry.

[CR42] Yang Y, Sheng G, Huang M (2006). Bioavailability of diuron in soil containing wheat-straw-derived char. Science of the Total Environment.

[CR43] Yu O-Y, Raichle B, Sink S (2013). Impact of biochar on the water holding capacity of loamy sand soil. International Journal of Energy and Environmental Engineering.

[CR44] Yu XY, Ying GG, Kookana RS (2006). Sorption and desorption behaviors of diuron in soils amended with charcoal. Journal of Agricultural and Food Chemistry.

[CR45] Zhang X, Sarmah AK, Bolan NS, He L, Lin X, Che L (2016). Effect of aging process on adsorption of diethyl phthalate in soils amended with bamboo biochar. Chemosphere.

