# Supplementary material for: Effect of Biochar Amendment and Ageing on Adsorption and Degradation of Two Herbicides
Source: Water Air Soil Pollut. 2017 May 25;228(6):216. doi: 10.1007/s11270-017-3392-7 (PMC5443863; doi:10.1007/s11270-017-3392-7)
Supplement: Supplementary file 1 — (DOCX 114 kb) [file 11270_2017_3392_MOESM1_ESM.docx]

**Supplementary material**


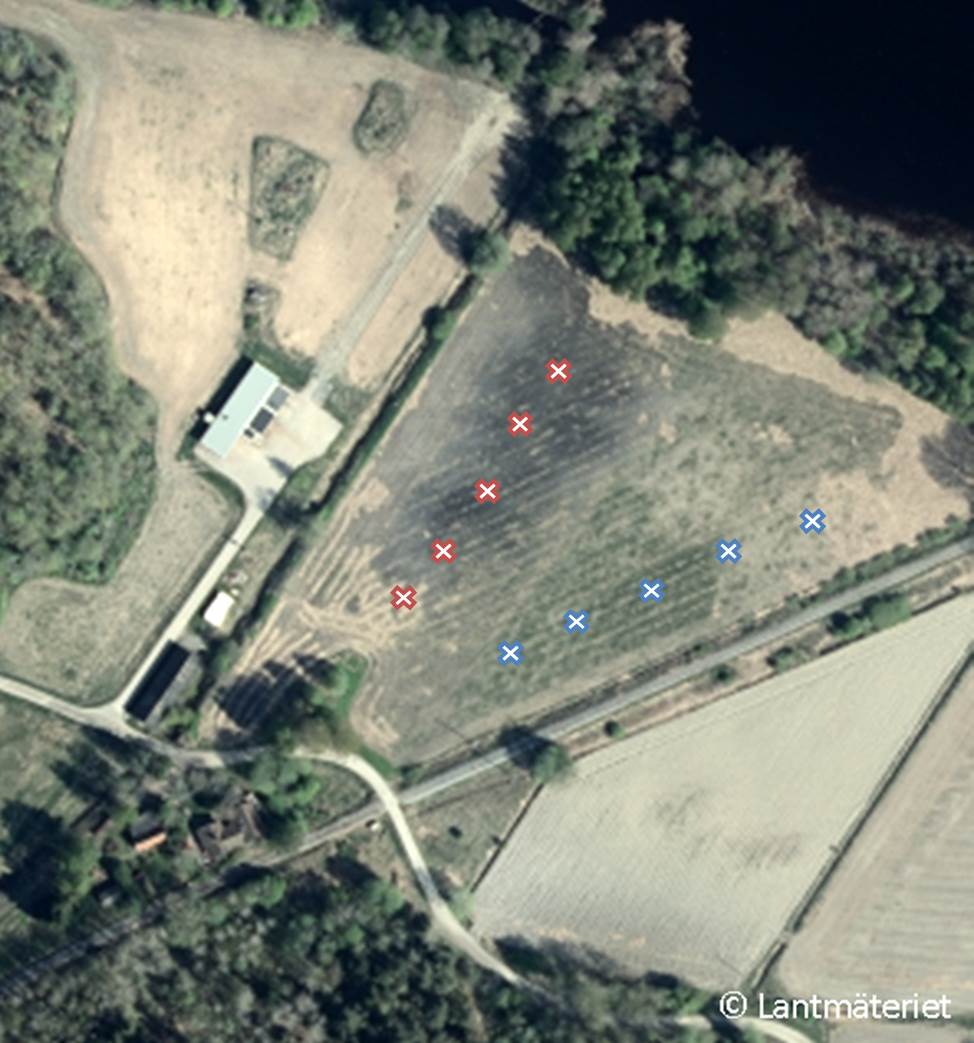


**Fig. S1.** Satellite image of Länna field. Blue crosses mark the sampling places for L, red crosses for LB.
