# Supplementary material for: Effect of Biochar Amendment and Ageing on Adsorption and Degradation of Two Herbicides
Source: Water Air Soil Pollut. 2017 May 25;228(6):216. doi: 10.1007/s11270-017-3392-7 (PMC5443863; doi:10.1007/s11270-017-3392-7)
Supplement: Supplementary file 2 — (DOCX 14 kb) [file 11270_2017_3392_MOESM2_ESM.docx]

**Supplementary Table 1** Some properties of the two herbicides used^a^

| Herbicide | Molecular mass  (g mol^-1^) | Water solubility  (mg L^-1^) | K_foc_ | log(K_ow_) | Dissociation constant (pKa) |
| --- | --- | --- | --- | --- | --- |
| Glyphosate | 169.1 | 10 500 | 16331 | -3.2 | 2.34, 5.73 |
| Diuron | 233.09 | 35.6 | 1067 | 2.87 | Not applicable |

^a^From the PPDB: Pesticides Properties Database (Lewis et al. 2016)
